# Long-Term Stability and Differentiation Potential of Cryopreserved cGMP-Compliant Human Induced Pluripotent Stem Cells

**DOI:** 10.3390/ijms21010108

**Published:** 2019-12-23

**Authors:** Mehdi Shafa, Tylor Walsh, Krishna Morgan Panchalingam, Thomas Richardson, Laura Menendez, Xinghui Tian, Sahana Suresh Babu, Saedeh Dadgar, Justin Beller, Fan Yang, Behnam Ahmadian Baghbaderani

**Affiliations:** Cell Therapy Process Department, Lonza Houston, Inc., Houston, TX 77047, USA; mehdi.shafa@lonza.com (M.S.); tylor.walsh@lonza.com (T.W.); krishna.panchalingam@lonza.com (K.M.P.); thomas.richardson@lonza.com (T.R.); laura.menendez@lonza.com (L.M.); xinghui.tian@lonza.com (X.T.); sahana.sureshbabu@lonza.com (S.S.B.); saedeh.dadgar@lonza.com (S.D.); justin.beller@lonza.com (J.B.); fan.yang@lonza.com (F.Y.)

**Keywords:** induced pluripotent stem cells, cryopreservation, stability, cGMP, differentiation, Telomere

## Abstract

The clinical effectiveness of human induced pluripotent stem cells (iPSCs) is highly dependent on a few key quality characteristics including the generation of high quality cell bank, long-term genomic stability, post-thaw viability, plating efficiency, retention of pluripotency, directed differentiation, purity, potency, and sterility. We have already reported the establishment of iPSC master cell banks (MCBs) and working cell banks (WCBs) under current good manufacturing procedure (cGMP)-compliant conditions. In this study, we assessed the cellular and genomic stability of the iPSC lines generated and cryopreserved five years ago under cGMP-compliant conditions. iPSC lines were thawed, characterized, and directly differentiated into cells from three germ layers including cardiomyocytes (CMs), neural stem cells (NSCs), and definitive endoderm (DE). The cells were also expanded in 2D and 3D spinner flasks to evaluate their long-term expansion potential in matrix-dependent and feeder-free culture environment. All three lines successfully thawed and attached to the L7^TM^ matrix, and formed typical iPSC colonies that expressed pluripotency markers over 15 passages. iPSCs maintained their differentiation potential as demonstrated with spontaneous and directed differentiation to the three germ layers and corresponding expression of specific markers, respectfully. Furthermore, post-thaw cells showed normal karyotype, negative mycoplasma, and sterility testing. These cells maintained both their 2D and 3D proliferation potential after five years of cryopreservation without acquiring karyotype abnormality, loss of pluripotency, and telomerase activity. These results illustrate the long-term stability of cGMP iPSC lines, which is an important step in establishing a reliable, long-term source of starting materials for clinical and commercial manufacturing of iPSC-derived cell therapy products.

## 1. Introduction

Pluripotent stem cells (PSCs) have the potential for long-term self-renewal and expansion while maintaining their differentiated potential. PSCs can be manufactured in large quantities in an undifferentiated state and be cryopreserved as master cell banks (MCBs) and working cell banks (WCBs) under current good manufacturing procedures (cGMPs) [1,2]. This unique characteristic of PSCs makes them an attractive source for regenerative cell therapy. These banks can be subsequently thawed, expanded, and differentiated for clinical applications. Establishing appropriate MCB and WBC strategies would allow access to high-quality starting materials that could minimize potential variabilities during the development of final PSC derived cell therapy products. In some instances, these starting materials might need to stay cryopreserved for many years after the initial cell manufacture/banking. Therefore, long-term cryopreservation of quality-assured PSCs is required to ensure an adequate supply of these cells for clinical and commercial manufacturing.

PSCs are often maintained, passaged, and cryopreserved as clumps of cells. Prior observations suggest that different cryopreservation solutions, cell lines, and cryopreservation strategies affect the revival of the cells post thaw and may lead to inefficient and slow cell revival [3]. Long-term storage of PSCs in LN_2_ carries a risk of mycoplasma, bacterial, and viral infection [4]. In addition, long-term storage may cause a loss of critical quality attributes (CQA) including identity, purity, potency, and sterility. Recently, several groups have raised concerns about critical cryoprotectant materials used in the stem cell cryopreservation such as Dimethyl sulphoxide (DMSO), fetal bovine serum (FBS), or serum replacement [5]. DMSO has been demonstrated to be toxic to the tissues and cells depending on the exposure time [3,6]. DMSO is also reported to cause cryoinjury including chromosomal/telomere instability and epigenetic changes in embryonic stem cells (ESCs) [7].

Owing to these concerns and in order to demonstrate the long-term stability of induced PSCs (iPSCs), we have evaluated the effect of long-term cryopreservation on the CQAs of human iPSC lines post thaw, including cell revival, 2D and 3D expansion, expression of pluripotency markers, spontaneous and directed lineage-specific differentiation, sterility, genomic stability, and changes in telomerase activity and telomere length. Our findings from this study can generate a more in-depth understanding of the impact of long-term cryopreservation on quality of human iPSC banks and their utility for the generation of high-quality clinical products.

## 2. Results

### 2.1. iPSCs Were Efficiently Thawed and Expanded in 2D Environment While Maintaining Pluripotency, Sterility, and Genomic Stability

**Figure 1:**
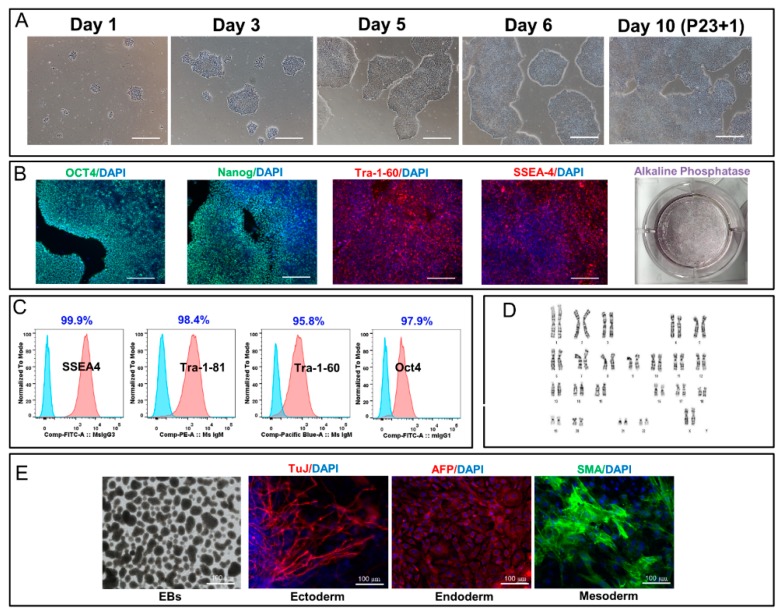
Thaw, expansion, and characterization of human LiPSC-18R post prolonged cryopreservation. (**A**) The induced pluripotent stem cells (iPSCs) attached and formed typical PSC colonies one day post thaw. The cells were passaged on day 6 and formed colonies one passage post thaw. Scale bars, 100 µm. (**B**) iPSCs stained positively with OCT4, TRA-1-60, SSEA4, NANOG, and AP. Scale bars, 100 µm. (**C**) iPSCs expressing the pluripotent stem cell internal and surface markers OCT4, SSEA4, TRA-1-60, and TRA-1-81 (dark pink). Light blue indicates the isotype control. (**D**) The iPSCs demonstrated a normal karyotype one passage post thaw. (**E**) iPSCs differentiated into embryoid bodies and readily expressing the markers for early ectoderm (TUJ1, beta-tubulin), endoderm (AFP, alpha feto protein), and mesoderm (SMA, smooth muscle actin). Scale bars, 100 µm.

Human iPSCs used in the current study were manufactured using the cGMP compliant manufacturing process and cryopreserved as previously reported [2]. All three lines were generated from healthy donors. The ethnicity and genetic background of the lines used in this study were summarized in Table S1. These studies were conducted on cell lines stored for five years in vapor phase of liquid nitrogen. Three iPSC lines (three vials each) of LiPSC-TR1.1, LiPSC-18R, and LiPSC-ER2.2 were thawed and the average cell count and viability (CCV) post thaw were measured. All three lines showed viability ranging from 75.2% to 83.3% with a percent recovery around 82.0% for LiPSC-18R and TR1.1 and 57.5% for LiPSC-ER2.2 (Table 1). The iPSC aggregates attached to L7^TM^ matrix-coated vessels and exhibited typical morphology of pluripotent cells between 6 and 8 days post plating (Figure 1A, Figures S1A and S2A).

Immunoflourescent staining of iPSC colonies showed typical PSC morphology with expression of pluripotency markers SSEA4, Tra-1-81, Tra-1-60, and Oct4 one passage post thaw. Akaline phosphatase (ALP) staining also confirmed the high efficiency of plating and revival efficiency (Figure 1B, Figures S1B and S2B). Flow cytometry analysis of the thawed iPSCs demonstrated that more than 95.0% of the cell population maintained their expression of pluripotency markers during long-term cryopreservation (Figure 1C, Figures S1C and S2C). All three lines showed normal karyotype post thaw without any obvious chromosomal aberrations (Figure 1D, Figures S1D and S2D). All nine thawed vials (3 lines) were negative for mycoplasma one day post thaw, at 5 passages, and passed sterility testing one day post thaw. All three undifferentiated iPSCs were harvested as aggregates and induced to form EBs in suspension culture. After 24 h, iPSCs formed clusters with compact appearance and various sizes. Suspended EBs were subsequently plated on gelatin-coated plates to induce further spontaneous differentiation to three lineages (Figure S3). Immunoflourescence staining of attached cells revealed positive cells from ectoderm (beta-tubulin, TuJ1), Endoderm (alpha feto protein, AFP) and Mesoderm (smooth muscle actin, SMA) (Figure 1E, Figures S1E and S2E). iPSCs were maintained for another 15 passages on static culture to demonstrate their cellular stability post thaw (Figure S4A). All three lines showed normal karyotype post 2D maintenance (Figure S4B).

### 2.2. Long-Term Cryopreserved iPSCs Maintain Their ECTODERM and Endoderm Lineages Directed Differentiation Potential

**Figure 2:**
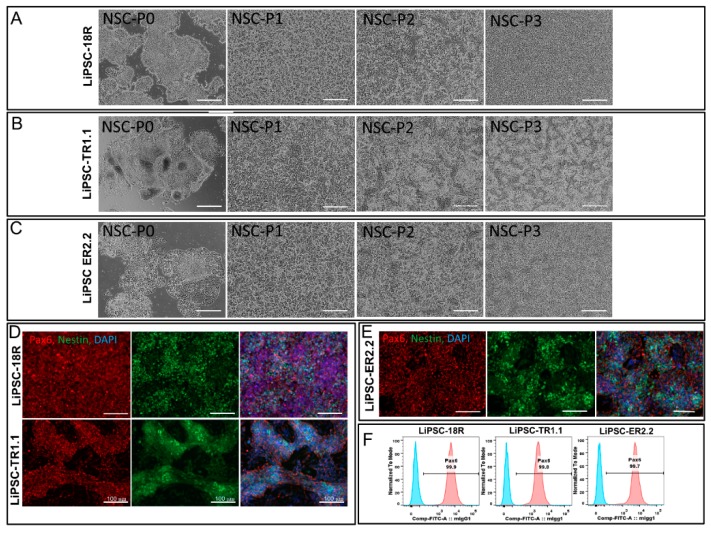
Neural differentiation of LiPSC lines one passage post thaw. (**A**–**C**) Morphology of LiPSC lines was changing during the differentiation from passage 0 (P0) to P3. (**D**–**E**) Immunofluorescent staining of neural stem cells (NSCs) at the end of P3 revealed that they express NSCs-specific markers (Nestin and Pax6). (**F**) Folw cytometry analysis at the end of P3 demonstrated high expression of Pax6 by cells acquired NSCs morphology. Scale bars, 100 µm.

In order to demonstrate the inherent directed differentiation potential of long-term cryopreserved iPSCs, LiPSCs-18R, LiPSC-TR1.1, and LiPSC-ER2.2 were induced into neural stem cells (NSCs, ectoderm) using a protocol originally established by Li and colleagues [8]. It has been shown that treatment of iPSCs with small molecules (CHIR99021 and SB431542) and LIF (leukemia inhibitory factor) induced the cells to primitive NSCs by modulation of transforming growth factor (TGF)-beta/Activin receptors [9] and canonical Wnt signaling (Glycogen synthase kinase, GSK3 inhibition) [10]. Some key optimizations and modifications were added in the protocol including Y-27632 inhibitor treatment time, optimized plating density during differentiation, replacement of feeders with a feeder-free culture system, and the basal media used. The cells differentiated to NSCs as shown by morphology changes and the appearance of epithelial/rosette-like structures formation and at the end of day 7 (P0) and during the entire process (Figure 2A–C). Differentiated cells were cultured and expanded for three weeks (three passages) and cryopreserved at the end of each passage. Immunofluorescent staining of Nestin and Pax6 (an early marker of neural induction) indicated the acquisition of NSCs phenotype at the end of passage 3 by the majority of iPSCs (Figure 2D–E). This observation was confirmed by flow cytometry analysis, which showed that more than 90.0% of cells expressed Pax6 on day 24 (P3) (Figure 2F). These uniformly differentiated neural cells were expanded on Poly-L-Ornithine/Laminin coated plates in the presence of SB431542, CHIR99021, and hLIF.

**Figure 3:**
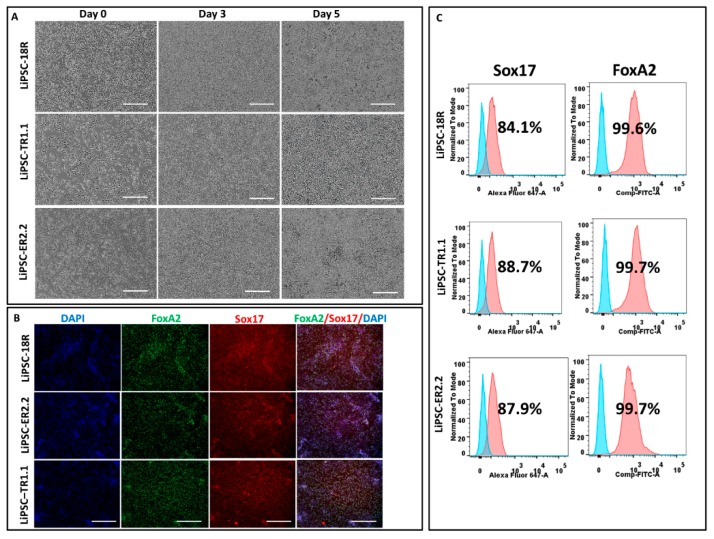
Differentiation of human iPSCs into definitive endoderm (DE) one passage post thaw. (**A**) Morphology of LiPSCs-derived cells was changing during the differentiation from day 1 to day 5. The cells were highly proliferative during the differentiated period and produced dense single-layered sheets. (**B**) Immunofluorescent staining of day 5 cells (all three LiPSC lines) revealed that they express DE-specific markers (FoxA2 and Sox17). (**C**) Flow cytometry analysis at the end of day 5 demonstrated that more than 80% of LiPSC 18R-derived cells express Sox17 and more than 99.0% express FoxA2. Scale bars, 100 µm.

iPSCs were differentiated to definitive endoderm (DE) lineage using a commercially available kit. Lower concentrations of Activin A induce mesodermal lineage differentiation [11], while a higher concentration at 100 ng/mL is generally used to induce iPSC differentiation to DE [12]. Activin A is produced by many cell types throughout development and is a member of the transforming growth factor beta (TGF-β) family of proteins. It has an important impact on the variety of cell functions including metabolism, apoptosis, and proliferation [13]. iPSCs’ morphology was changed until day 5 (Figure 3A). Immunofluorescent staining of endoderm markers (FoxA2 and Sox17) [14] was used to confirm the differentiation of iPSCs to DE for all three iPSC lines (Figure 3B). More than 80% of LiPSC-18R derived cells were positive for Sox17 and FoxA2 as specific markers for DE on day 5, as shown by flow cytometry analysis (Figure 3C).

### 2.3. Long-Term Cryopreserved Human iPSCs Retain the Capacity to Differentiate into Cardiac Lineage under Defined Conditions

**Figure 4:**
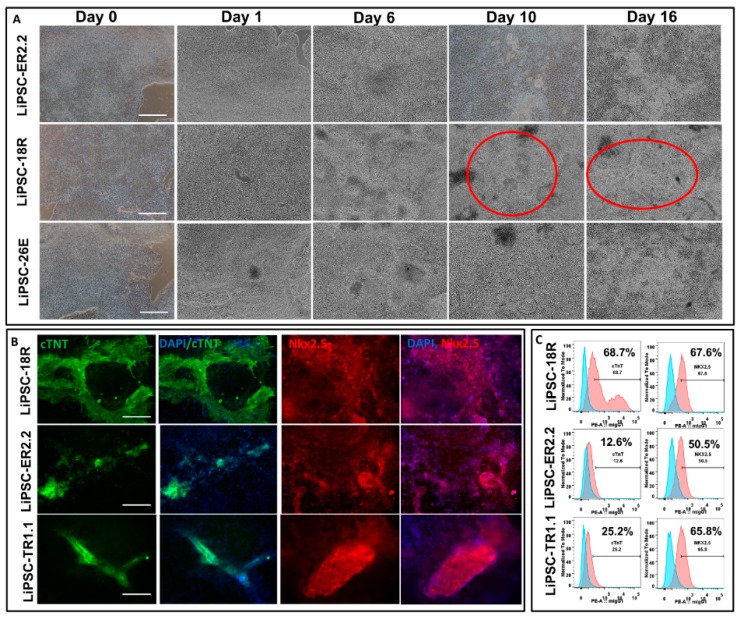
All three current good manufacturing procedure (cGMP)-compliant LiPSCs lines differentiated into cardiomyocytes (CMs) in two-dimensional (2D) culture system. (**A**) Starting with good quality cells (around 100% confluency with very low differentiated areas), three LiPSC lines started morphological changes from day 1 onward until day 14. The first beating was observed for two lines (LiPSC-18R and Er2.2) around day 6–7 within several patches. A large beating area was shown for LiPSC-18R on day 10 and 14 (red circles). (**B**) All of the beating areas on day 14 of differentiation were positively stained for cardiac-specific troponin (cTnT) and Nkx2.5 markers for three LiPSC lines. (**C**) Three LiPSC lines showed positive population expressing NKX2.5 and cTnT. LiPSC-18R showed the highest population expressing NKX2.5 and cTnT among the three. Scale bars, 100 µm.

Differentiation of three iPSC lines into cardiomyocytes (CMs) was performed over 14 days in 2D using established protocols [15]. During the differentiation state, the morphology of the cells rapidly changed during the Wnt pathway induction and inhibition (day 0–4) and continued until day 14 (Figure 4A). For LiPSC-18R and LiPSC-ER2.2 lines, the first cell beating appeared on day 7 or 8 post-induction. No beating patches were observed for LiPSC-TR1.1 during the differentiation. For liPSC-18R, the beating cells were initially confined to a small area, but expanded gradually to various sizes (ranging from 500 µm to a few millimeter) of synchronized beating areas, but for the LiPSC-ER2.2, there was only a limited number of beating areas and these areas were small (Figure 4A and Supplementary Videos S1 and S2). Immunofluorescent staining of the differentiated cells on day 14 showed expression of cardiomyocyte specific markers including cTnT and Nkx2.5 (Figure 4B). Flow cytometry analysis of the iPSC-derived CM further demonstrated cTnT and Nkx2.5 expression. The expression of Nkx2.5 on day 14 ranged between 12.6% (for LiPSC-ER2.2) and 68.7% (for LiPSC-18R). The expression of cTnT on day 14 post induction ranged between 50.5% (for LiPSC-ER2.2) and 67.7% (for LiPSC-18R). Viability of harvested cells on day 14 was more than 90% and each well of a six well plate generated around 2.5 × 10^6^ viable cells for each line.

### 2.4. iPSCs Were Efficiently Thawed and Expanded in 3D Environment While Maintaining Pluripotency and Spontaneous Differentiation

**Figure 5:**
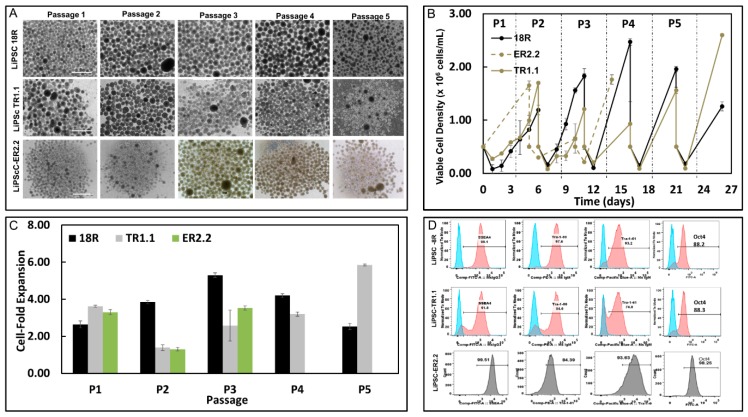
All three cGMP compliant LiPSCs lines were expanded in 3D suspension culture environment while maintaining their pluripotency. (**A**) Photomicrograph of the human PSCs (hPSCs) in 3D suspension culture during five passages in the Biott bioreactor. (**B**) Viable cell density during five pasages in Biott suspension bioreactors. (**C**) Three lines showed different cell-fold expansion during 3D culture. (**D**) All three lines demonstrated a high level of pluripotency specific markers: SSEA4, Tra-1-81, Tra-1-60, and Oct4 post expansion in the Biott bioreactor. Scale bars, 100 µm.

For most transplantation therapies requiring derivatives of human iPSCs, it is necessary to implement culture strategies that can be scaled-up efficiently. One method is the use of stirred-tank bioreactors, where the cells can be expanded and differentiated as free-floating aggregates in 3D. In this study, we evaluated the ability of the cryopreserved iPSCs lines to expand in this manner in 3D by demonstrating that the cells expanded over three (LiPSC ER2.2) and five (LiPSCTR1.1 and LiPSC-18R) passages in suspension culture (Figure 5A). Over each passage, cell-fold expansion of 2.0–5.9-fold was observed while maintaining their genomic stability and pluripotency (Figure 5B–D and Figure S5A). All three lines had variable fold expansion that might require optimization of the culture conditions—for example, culture medium and hydrodynamic environment. Three LiPSCs lines were induced to form embryoid bodies (EBs) post Biott expansion in order to show their potential of three-lineage differentiation. All three lines formed typical EB aggregates during the 10 days in suspension and developed cell outgrowth after plating on gelatin-coated plates (Figure S5B).

### 2.5. Passaged iPSCs Showed Reduced Telomere Length with Higher Telomerase Activity Compared with Freshly-Thawed Cells

**Figure 6:**
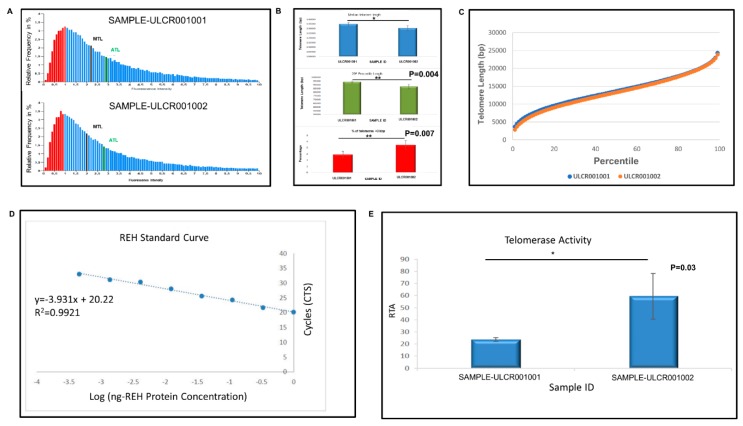
Distribution of telomere lengths in fresh and passaged iPSCs. (**A**) Bars represent the relative frequency for every particular fluorescence intensity normalized (X axis). The 20th percentile (red bars) indicates the particular length below which 20% of the telomeres were observed. The median (MTL) and average telomere length (ATL) are also indicated in the histogram. The relative frequency distribution along the X axis allowed for the analysis of telomere length variability. (**B**) Telomere analysis technology (TAT) results (median telomere length, 20th percentile telomere length, and % short telomeres) for both samples. (**C**) Comparison of percentile curves for both samples. (**D**) Standard curve of Ct values of protein from cells against log of protein (ng). The cycle number at the threshold (Ct value) for each sample was interpolated in the curve in order to calculate relative telomerase activity (RTA). (**E**) Quantitative telomeric repeat amplification protocol (Q-TRAP) results (RTA) for two samples.

Telomere length and telomerase activity was compared between the cryopreserved LiPSC-18R (directly after thaw) and the cells after 15 passages in 2D adhesion culture. Both samples recovered with over 70.0% viability (Table 2). Cryopreserved cells showed higher telomere length percentile values compared with the cells that underwent 15 passages (Figure 6A, Table 2). Cryopreserved iPSC appeared to have a lower percentage of short telomeres below 10,500 bp, as well as a higher percentage of long telomeres above 12,000 bp compared with the passaged cells. A reduction of long telomeres and an increase in short telomeres were also observed from freshly thawed cells when compared with cells after fifteen passages. Freshly thawed-cells showed lower percentage of cells with short telomeres (median < 12,500 bp) and a greater percentage of cells with long telomeres (median > 15,000 bp) than the passaged cells (Figure 6A,B).

When we compared the cells using quantitative telomeric repeat amplification protocol (Q-TRAP) for the telomerase expression, the results were the opposite. The protein concentration in freshly thawed and 15 passages was 17.9 and 42.7 µg, respectively. The regression curve’s coefficient of determination (REH cell line-Human Acute Lymphocytic Leukemia) was >0.9 (*R^2^* = 0.9971) and telomerase activity was found to be significantly higher in passaged cells compared with freshly-thawed iPSCs (Figure 6B).

## 3. Discussion

We have previously reported the development of a manufacturing process to generate cGMP-compliant human iPSC lines with detailed characterization of the generated cell lines [1,2]. Large-scale manufacturing of cGMP-iPSC banks is a key step towards the establishment of a reliable starting material for regenerative medicine products. It requires that these banked cells maintain their critical quality attributes post thaw and their ability to generate functional, therapeutically relevant cell products. The effectiveness of cryopreserved stem cells from different sources, including bone marrow and cord blood, has been demonstrated for several disorders that include, but are not limited to, graft versus host disease [15,16], Scleroderma [17], Thalassemia [18], and multiple sclerosis [17,19]. Implementing a successful cryopreservation strategy can stabilize the supply of critical therapeutic products and support centralized manufacturing operations.

To date, the primary focus of academic and industrial labs has been mainly on the characterization of undifferentiated human iPSC lines post-derivation and expansion rather than post-cryopreservation. Despite the implementation of cryopreservation as a routine and conventional method for preserving iPSCs long-term, there is limited knowledge on how the cryopreservation and thaw strategy affects the iPSC genomic integrity and differentiation capacity to desired lineages. Some groups have shown that freeze/thaw process may lead to DNA and chromosomal aberrations due to production of free radicals in some cell types [20], but to the best of our knowledge, there is no such study on the long-term stability of cryopreserved iPSC MCBs and/or WCBs.

Our data showed that, after five years of cryopreservation, all three cGMP-manufactured cell lines demonstrated normal karyotypes post thaw. The lines maintained their genomic integrity for 15 passages in 2D culture environment and for 5 passages in 3D suspension culture. Several groups have demonstrated that cryopreservation and recovery of human ESCs trigger apoptosis, loss of pluripotency, and spontaneous differentiation [21,22]. The recovery of human ESCs decreases to 16%–23% during the freeze/thaw process, as measured by the number of attached colonies 9–14 days post thaw combined with a low growth rate and high percent of differentiation [23,24]. Our results indicated that all three lines could be thawed efficiently with high plating rate within 7–9 days with less than 5.0% differentiation observed. The plating rate was demonstrated by measuring the attachment efficiency (number of iPSC colonies attached after thaw/passaging) and detected through alkaline phosphatase staining. Although the viability of one line (ER2.2) was approximately 58.0% post thaw, all three lines exhibited a high level of attachment and formed typical PSCs colonies in 7–9 days prior to the first passage. Also, all three lines preserved their critical quality attributes (CQA) including spontaneous and directed differentiation potential to cells from the ectoderm, mesoderm, and endoderm lineages after prolonged cryopreservation. These lines were fully characterized for their CQA prior to the cryopreservation five years ago, as demonstrated in our previous reports [1,25]. As the directed differentiation has not been optimized for each line, the three lines showed variability in their differentiation capacity. Line-to-line differentiation variability may depend on several factors including chromatin status, DNA methylation signature (somatic memory), reprogramming method, and tissue source [26,27]. Stability of human iPSC lines to proliferate and expand in 3D suspension environment post-prolonged cryobanking is also one of the key factors for the generation of a large-number of cells to be derived into clinically-relevant cells in the regenerative medicine field [28,29]. We evaluated all three lines for their inherent potential to form free-floating aggregates (i.e., no microcarriers), self-renew, and have the ability to expand up to clinically-relevant cell density in stirred suspension bioreactors. Our results showed that long-term cryopreservation of these lines did not affect their expansion potential in 3D culture. Furthermore, all three lines maintained their CQA such as pluripotency and differentiation capacity and showed normal karyotype after five passages.

Pluripotent stem cells show high telomere length and appropriate telomerase activity to support their self-renewal and proliferation capacity [30,31]. Furthermore, telomere maintenance, regulation, and homeostasis is critical for hPSC long-term culture and its chromosomal stability [31]. LiPSC-18R line (+15 passages) demonstrated higher telomerase expression (reverse transcriptase TERT and RNA template TERC) and lower telomere length compared with the freshly-thawed cells that were cryopreserved five years ago. The shorter telomere length observed after 15 passages may be caused by cell division during the early passages post thaw. To compensate for this shorter telomere length and to maintain the pluripotency and chromosomal integrity, human iPSCs induce the expression of telomerase to extend the telomere length as evident by the higher telomerase activity following 15 passages post thaw. In agreement, Zeng et al. demonstrated that telomeres were elongated during in vitro expansion and were then stabilized through a telomerase-dependent mechanism [32]. Our 2D and 3D expansion and directed differentiation of the revived cells demonstrated stable expression of telomerase and telomere length, as several studies have reported a correlation between short telomeres and unstable directed differentiation in mouse and human pluripotent cells [33,34].

The effectiveness of human iPSCs in research and therapeutic applications highly relies on their genomic, phenotypic, and molecular stability during long-term passaging, expansion, and prolonged cryopreservation. This study addresses these aspects and the stability of cryopreserved GMP-manufactured iPSCs after storage in LN_2_ for five years. Considering the number of iPSC lines assessed (*n* = 3) in the current study, more lines that are generated with different reprogramming methods need to be evaluated in the future studies. In conclusion, this study suggests that iPSCs remain stable and therapeutically-efficacious after banking and that the current cryopreservation strategy can be utilized for the long-term storage of iPSCs.

## 4. Materials and Methods

### 4.1. Thawing and Cell Culture

A cGMP-compliant environment was used to generate the iPSC lines LiPSC-18R, LiPSC-TR1.1, and LiPSC-ER2, as described before [2], and continuously maintained on the defined L7^TM^ hPSC matrix (Lonza, FP-5020) using L7™ hPSCs medium. The L7^TM^ hPSC medium consists of basal L7^TM^ hPSC medium (FP-5107) and L7^TM^ hPSC supplement (FP-5207). Per the manufacturer’s protocols, the cells were serially passaged using L7^TM^ hPSC passaging solution (FP-5013) and cultured in humidified a 37 °C incubator at 5% CO_2_. These lines were cryopreserved under cGMP condition in CryoStor CS10 (BioLife Solutions, Bothell, WA) with a cell density of 1.0–2.0 × 10^6^/mL (Table 1) and maintained for five years in the vapor phase of LN_2_. Briefly, three vials for each iPSC line were thawed in a 37 °C water bath for 3–4 min and the content of each vial was transferred to a 15 mL conical tube. Then, 9 mL of L7^TM^ hPSC medium was added drop by drop to the cells to minimize the osmotic shock to the cells. The cell suspension was centrifuged at 300× *g* for 5 min. The supernatant of the cells was removed and the cells were resuspended in 2–3mL of L7^TM^ hPSC medium. The cells were plated on L7^TM^ matrix coated plates using L7^TM^ hPSC medium. Total cell count and viability was measured before plating using the NucleoCounter NC-200 (Chemometec, Denmark) with Via1-cassettes (two cassettes count with Solution 10). Vials from each line were pooled together and plated on at least six wells. All thawed cells were maintained undifferentiated for 15 passages in a 2D culture system followed by 5 passages in a 3D culture system. Cells in 2D culture were passaged as clumps in static culture every 4–6 days utilizing a ratio between 1:6 to 1:12 based on the confluency.

### 4.2. Post-Thaw Expansion of iPSCs in 3D Biott Bioreactor System

Each of the iPSC lines were harvested from the 2D cultures after 3–4 passages as single cells and inoculated in ABLE Biotts (Reprocell, Beltsville, MD) in either L7^TM^ or Nutristem, in duplicate, at a cell density of 5.0 × 10^5^ cells/mL in a volume of 30 mL. On Day 0, the culture medium was supplemented with 10 µm Y-27632. The Biotts were agitated at 60 rpm and placed in a humidified incubator operating at 37 °C and 5% CO_2_. Every day, a 50% medium change was performed by removing the Biotts from the incubator and allowing the aggregates to settle for 5 min. After 5 min, 50% of the medium was removed and centrifuged at 200× *g* for 3 min to collect any aggregates in suspension. After centrifugation, the supernatant was discarded, the collected aggregates were resuspended in fresh medium and added back into the appropriate bioreactor. The Biotts were fed until the cells reached between 1.5 × 10^6^ and 2.0 × 10^6^ cells/mL.

When the Biotts reached this cell density, the culture was removed from the incubator, and the cell aggregates were collected and centrifuged at 200× *g* for 3 min. The supernatant was discarded, and the aggregates were washed one time with 1 × phosphate buffer saline (PBS), and centrifuged again at 200× *g* for 3 min. The supernatant was discarded, and the aggregates were enzymatically dissociated to single cells in the presence of TrypLE. The TrypLE-cell aggregate solution was placed for 5 min in a 37 °C water bath, and the culture was triturated with a P1000 five times to break up the aggregates. The enzymatic reaction was diluted with culture medium and the cell suspension was centrifuged at 300× *g* for 5 min. Following centrifugation, the supernatant was discarded, the cells were resuspended in the appropriate culture medium + 10 µm Y27632 and re-inoculated at a density of 5.0 × 10^5^ cells/mL. This process was repeated for a total of five passages, after which the cells were evaluated for spontaneous differentiation and karyotype.

### 4.3. Cell Counts

Cell counts were performed daily for passage 1 (P1) and P2 to determine the growth kinetics. For P3–5, cell counts were only done on day 1 and on harvest day (as determined by the counts during P1/2). To obtain the cell counts, a 1 mL sample was removed from the Biott and placed in a 15 mL conical tube and centrifuged at 200× *g* for 3 min. The supernatant was discarded and the cells were washed with 1 × PBS, and centrifuged at 200× *g* for 3 min. Following centrifugation, the supernatant was discarded and the cells were resuspended in TrypLE and placed in a 37 °C water bath. After 5 min, the cells were pipetted to break apart the aggregates, culture medium was added to the suspension to dilute the enzymatic reaction, and then centrifuged at 300× *g* for 5 min. The supernatant was discarded and the cells were then resuspened in culture medium and counted using the two-cassette method on the NC-200 (i.e., Solution 10 method).

### 4.4. Flow Cytometry

Flow cytometry was performed on the iPSCs when they reached approximately 70% to 80% confluency at P1 and P15 post thaw in 2D. Flow cytometry analysis was also performed on samples taken at five passages post thaw on iPSCs cultured in a 3D culture system. The iPSCs were dissociated into a single-cell using TrypLE solution (ThermoFisher). Then, 4% paraformaldehyde (PFA) and perm/wash buffer (Becton Dickinson) were used to fix and permeabilize the cells for intracellular staining respectively. Cells were incubated with Alexa Flour 488 anti-IgG isotype control and Alexa-488 anti-OCT3/4 (Cell Signaling, 5177S) post permeabilization. Prior to fixation, PE-conjugated antigen-specific antibodies and respective isotypes with manufacturer’s recommended concentrations were used to detect membrane antigens as below: anti-SSEA4 (Becton Dickinson, 560128), anti-TRA-1-60 (Becton Dickinson, 560193), anti-TRA-1-81 (Becton Dickinson, 560161), anti-IgM isotype (Becton Dickinson, 555584), and anti-IgG3 isotype (Becton Dickinson, 556659). FACSCanto™ II flow cytometer (Becton Dickinson) was used to process the samples. BD FACS Diva and Flowjo 7.6 softwares were used to acquire flow data and analyze the results.

iPSC-derived differentiated cells were harvested and dissociated to single cells. The cells are stained as follows: PBS (−/−) and Accutase solution (Millipore, SCR005) were used to wash and dissociate iPSC-derived neural stem cells (NSCs) into single cells, respectively, as per the manufacturer’s instruction (6 min at 37 °C). To quench the activity of Accutase, an equal volume of neural medium, supplemented with 10 µm Y-27632 (Abcam, ab120129) was added to the cells. Cells were fixed and permeabilized for intracellular staining with 4% PFA and perm/wash buffer (Becton Dickinson, 554723) for post cell count and viability measurement, respectively. Alexa Fluor 488 mouse anti-Pax6 (BD, PN561664) or respective Alexa Fluor 488 anti-IgG2a isotype control (BD, PN558055) were used to stain permeabilized cells. iPSC-derived DE cells were harvested as single cells using TrypLE and stained with Sox17 and FoxA2, as described above.

A mixture of Liberase (2.5 mg or 13 units/mL) and TrypLE solution (50:1) was used to digest iPSC-derived cardiomyocytes for 15 min at 37 °C post PBS (−/−) wash. The solution was quenched with the same volume of cardiac differentiation medium. The cells were filtered using 100 µm cell strainer after 3–4 times trituration, and were fixed and permeabilized with the 4% PFA and perm/wash buffer, respectively, for intracellular staining. Anti-cTnT (ab8295) or respective anti-IgG isotype control (abcam, ab91353) were used to stained the permeabilized cells. For the second antibody, PE goat anti-mouse IgG (ThermoFisher, P21129) was used (ThermoFisher, P21129) to stain the cells post wash. The positively stained cells were then captured through a FACSCanto™ II flow cytometer (Becton Dickinson). Flow data were acquired using BD FACS Diva software and then analyzed with Flowjo 7.6 software.

### 4.5. Immunocytochemistry

iPSCs and derived-differentiated cells were stained as per the following method: the cells were washed twice with 1 × Dulbecco’s phosphate buffered saline (DPBS) (Lonza Biosciences, 17-513F) post culture medium aspiration. The washed cells were then fixed with 4% PFA (Electron Microscopy Sciences, 15710) for 20 min. The cells were permeabilized with 0.1% Triton X-100 (Sigma-Aldrich, T9284) for 15 min and blocked with 10% goat serum in PBS (−/−) in 0.1% Triton X-100 at RT for 15 min. The primary antibodies were prepared in 1% bovine serum albumin (BSA) in 1 × DPBS and the cells were incubated overnight at 4 °C. Primary antibodies against NANOG (R&D Systems, AF1997; 6.7µg/mL) and OCT4 (Abcam, ab19857; 1:350), Desmin (Dako, 1:300), Actin (Sigma, A111, 1:500), cTnT (abcam, ab8295, 1:200), FoxA2 (abcam, ab108422, 1:300), MYL2 (Sigma, HPA019763, 1:200), Pax6 (BioLegend, 901301, 1:300), Sox17 (R&D systems, MAB19241), and Nestin (R&D systems, MAB1259, 1:50) were used in combination with the related secondary antibodies Alexa Fluor 594 Goat anti mouse (ThermoFisher, A11005, 1:1000) and Alexa Fluor 488 Goat anti Rabbit (ThermoFisher, A11070, 1:1000). Cells were then rinsed three times with 1% BSA in 1x DPBS. All cells were incubated with 5 µg/mL 4’, 6-diamidino-2-phenylindole (DAPI) (ThermoFisher, D1306) in 1% BSA in 1x DPBS and secondary antibodies for 1 h at room temperature (RT) in the dark. PSCs (both iPSCs and ESCs as control) were treated with primary antibodies detecting surface markers TRA-1-81 (Stemgent, 09-0011; 1:100), TRA-1-60 (Millipore, MAB4360; 1:100), and SSEA4 (Millipore, MAB4304; 1:100) overnight at 4 °C post blocking step and prior to being permeabilized. All fluorescence images were captured using a Zeiss Observer.Z1 microscope equipped with ZEN software.

### 4.6. Embryoid Body (EB) Differentiation

iPSCs cultures were dissociated using L7^TM^ hPSC passaging solution as small aggregates. EB formation medium consisting of DMEM/F12 (Life Technologies, 11330-032) and 10 μM Y27632 (Millipore, SCM075) was used to resuspend the cell aggregates. Cell clumps were allowed to settle down by gravity in a conical tube. The supernatant was then removed and the cells were resuspended in fresh EB medium. Cell aggregates were plated on ultra low attachment plates (Corning, YO-01835-24) with a split ratio of 1:1 and incubated for 12 to 24 h. Large cell aggregates were collected into a conical tube after the incubation time and allowed to settle down by gravity. The medium was then replaced with differentiation medium (80% DMEM High Glucose (Life Technologies, 11965-092), 55 μM β-Mercaptoethanol (Life Technologies, 21985-023), 2 mM L-glutamine (Cellgro/Mediatech, 25-005-CI), 20% defined fetal bovine serum (FBS, Hyclone, SH30070.03), and 1x non-essential amino acids (NEAA, Life Technologies, 11140-050). The cell aggregates were then cultured in ultra low attachment plates using a split ratio of 1:1 in 0.4 mL differentiation medium/cm^2^. The differentiation culture medium was changed every other day for six days. EBs were seeded on gelatin-coated plates (EmbryoMax^®^ ES Cell Qualified Gelatin Solution (Millipore, ES006-B) on the seventh day at approximately 10 EBs/cm^2^. The EBs were allowed to attach to the coated dishes for two days. The medium was changed every other day afterward with 0.4 mL/cm^2^ differentiation medium. On day 14, spontaneously differentiated iPSCs were fixed and permeabilized with 4% PFA and 0.1% Triton X-100 PBS solution, respectively, as described above. The cells were incubated with DPBS containing 10% goat serum (Life Technologies, 10000C) for 2 h at RT post rinsing with PBS/Tween solution. Primary antibodies detecting beta-III-tubulin (Millipore, MAB1637; 1:400), smooth muscle actin (DAKO, M0851; 1:500), and alpha-1 fetoprotein (Abcam, ab3980; 1:200 or R&D systems, MAB1369, 1:100) were added to blocked cultures and incubated overnight at 2–8 °C. The cells were washed twice with PBS/Tween solution, and the secondary antibodies including Alexa Fluor 488-conjugated goat anti-mouse IgG(H+L) (Life Technologies, A11001; 1:1000) and Alexa Fluor 494-conjugated goat anti-mouse IgG (H+L) (Life Technologies, A-11032; 1:1000) and DAPI solution were added and incubated for at least 2 h at RT. The cultures were then rinsed three times (5 min each) in 1x DPBS and maintained in 50% glycerol for analysis.

### 4.7. Coating Protocol for Culturing NSCs

In order to prepare the vessels for the NSCs differentiation, each well of a six well plate was coated with 1 mL/well of poly-L-Ornithine (Sigma, P4957) diluted solution (20 µg/mL final concentration). The plates were incubated at 37 °C for 2 h or at 4 °C overnight. The wells were then rinsed twice with DPBS (−/−). Human recombinant Laminin (Biolamina, LN521) solution was added to each well (1 mL/well) at 15 µg/mL concentration and incubated at 37 °C for at least for 1 h, but not for more than 6 h. The Laminin solution was removed prior to plating the iPSCs.

### 4.8. Neural Stem Cell Differentiation

Cells were passaged from 2D culture using L7^TM^ passaging solution in order to generate small clumps. Cells were plated with L7^TM^ medium onto L7^TM^ matrix coated plates at a 1:10 to 1:20 ratio depending on the harvest cell density. After 16–20 h, the medium was switched to Lonza neural induction medium (3 mL/well of a six well plate, day 0) consisting of primary neuron basal medium (PNBM, Lonza, CC-3256), 1x B27 (ThermoFisher, 17504044), 1x Glutamax (ThermoFisher, 35050-061), 4 µm CHIR99021 (Tocris, 4423), 3 µm SB43152 (Stemgent, 04-0010), and 10 ng/mL human leukemia inhibitory factor (hLIF, Millipore, LIF1010). The media was changed every other day until day 7. The cells were passaged by exposing cells to Accutase for 8–10 min. Y27632 (5 µm/mL) was added to the neural induction medium after plating. The cells were then plated at 1.0 × 10^6^/well in a six well plate that was pre-coated with poly-L-ornithine/Laminin (NSCs-P1). On the following day, the medium was changed using neural induction medium (no Y27632). The medium was changed every other day until cells reached 95%–100% confluency (4–5 days). The cells were then passaged as described above and re-inoculated into new pre-coated (Poly-L-Ornithine/Laminin) wells of a six well plate. Further expansion of the cells was conducted to enable flow cytometry and immunostaining at P3 and cryopreserved at P4.

### 4.9. Definitive Endoderm Differentiation

iPSCs were induced to differentiate to definitive endoderm (DE) for five days using the STEMdiff Definitive Endoderm kit (Stem Cell Technologies, 05110) with some modifications to the manufacturer’s protocol: (1) All iPSC lines were cultured for 3–4 passages using L7^TM^ medium + matrix prior to differentiation. (2) The gentle cell dissociation reagent (Stem Cell Technologies, Cat#07174) was replaced with TrypLE to prepare the single cell suspension for day 0 of the protocol. (3) The iPSCs were plated at a density of 2.0 × 10^6^ cells/well onto pre-coated plates with L7^TM^ matrix to have a confluency of near 100% on day 1. The manufacturer’s instructions were followed for the rest of the protocol.

### 4.10. Differentiation of iPSCs to Cardiomyocytes

With small modifications to protocols described by Burridge et al. (Burridge, Matsa et al. 2014), iPSCs were induced to differentiate into cardiomyocytes (CMs). Prior to the initiation of differentiation, the iPSCs were first maintained in Lonza L7^TM^ medium + L7^TM^ matrix for 2–3 passages. On day 0 of differentiation, the cells were at approximately 95%–100% confluency in six well plates with minimal spontaneous differentiation (i.e., <5%) before the start of CM induction. On day 0 of the CM induction, the medium was changed to cardiac differentiation media (CDM) consisting of RPMI1640 basal media (Lonza, 12-702F). The basal media was supplemented with 213 μg/mL l-ascorbic acid 2-phosphate (Sigma, A8960-5mg), 500 μg/mL O. Sativa-derived recombinant human albumin (Sigma, A0237) and 6 µm CHIR99021 (Tocris, 4423) to complete the differentiation media. The cells were placed in a humidified incubator operating at 37 °C and 5% CO_2_. On day 1, the medium was completely exchanged with the same fresh medium containing CHIR99021. On day 2, the medium was replaced with CDM supplemented with 2 µm Wnt-C59 (Tocris, 5148), and a complete medium exchange was performed on day 3 with the same medium (i.e., CDM + Wnt-C59). On day 5, 6, and every other day up to day 14, a complete medium exchange was performed with CDM (3 mL/well on day 5 and 6; 2 mL/well from day 8 to 14). Spontaneous contractile activity first appeared around day 6–7 on different areas of the cell culture depending on the cell line. Representative images and videos were taken using Nikon Eclipse Ti inverted microscope using NIS Elements software.

### 4.11. Alkaline Phosphatase Staining

Alkaline phosphatase (AP) staining was performed using the Millipore kit (Cat#SCR066) according to manufacturer’s protocol.

### 4.12. Karyotype Analysis

Karyotype and Short Tandem Repeat (STR) analyses were performed by a qualified service provider (LabCorp, Santa Fe, MN) using standard G-banding methods.

### 4.13. Mycoplasma and Sterility Test

Mycoplasma testing was performed using MycoAlert Mycoplasma detection Kit (Lonza, LT07–118) as per manufacturer’s protocol. The supernatant of the cells was examined one day post thaw and after five passages in the 2D culture system. The sterility test was performed using Bac-T Alert sterility test following the manufacturer’s protocol.

### 4.14. Telomere Size and Telomerase Activity

Telomerase relative activity was measured by quantitative telomeric repeat amplification protocol (Q-TRAP) (Life Length, Madrid, Spain). Briefly, protein extracts were prepared by lysing the cell pellets and quantifying the protein concentration form the resulting lysates. Telomerase protein extracts were then incubated with a specific oligonucleotide substrate to allow the enzymatic addition of telomeric DNA repeats by the endogenous telomerase. Following the enzymatic reaction, telomerase extension products were amplified and quantified by real-time quantitative PCR (qPCR). In real time PCR, a positive reaction was detected by accumulation of a fluorescent signal. The C_t_ (cycle threshold) was defined as the number of cycles required for fluorescence to cross the threshold (i.e., exceeds background levels). The telomerase-positive standard dilution series was plotted against the telomere protein concentration (r^2^ > 0.9) as a standard curve of C_t_ values. The assay was performed in triplicate. The mean and standard deviation (SD) from each triplicate was calculated, which include both positive and negative controls. Data were reported as RTA (relative telomerase activity).

Telomere median length was determined by telomere analysis technology (TAT) using a high throughput Q-FISH (fluorescence in situ hybridization) technique (Life Length, Madrid, Spain). This method was modified for cells in interphase. In brief, telomeres were hybridized with a fluorescent peptide nucleic acid probe (PNA) that recognizes three telomere repeats (sequence: Alexa488-OO-CCCTAACCCTAACCCTAA, Panagene). Using a high-content screen system, the images of the nuclei and telomeres were captured. The length of the telomere was proportional to the intensity of the fluorescent signal from the PNA probe. Using a control cell line with known telomere length, the fluorescence intensities were translated to base pairs through a standard regression curve.

Sample preparation and Q-FISH: the samples and control cell lines were removed from liquid nitrogen storage and thawed in a water bath at 37 °C and counted to determine the cell number and viability.

Cells were seeded at a density of 15,000 cells/well in clear bottom black-walled 384-well plates. Replicates of each sample (five replicates) and each control cell line (five replicates) were seeded and fixed with methanol/acetic acid (3/1, vol/vol).

Once the cells are fixed onto the plate, they were treated with pepsin to digest the cytoplasm and the resulting nuclei are processed for in situ hybridization with the PNA probe. Following washing of the hybridized nuclei, the DNA was stained using a standard DAPI incubation, and the wells were subsequently filled with mounting medium and the plate was stored overnight at 4 °C.

HT Microscopy: the Acapella software, Version 1.8 (Perkin Elmer), was used to acquire and analyze quantitative images on a high content screening opera system (Perkin Elmer). The images for analysis were captured using a 40 × 0.95 NA water immersion objective. DAPI was detected using UV wavelengths and Alexa Flour 488 signals were detected using 488 nm wavelengths. With constant exposure settings, 15 independent images were captured at different positions for each well. Next, the nuclei images were used to define the region of interest for each cell, and the telomere fluorescence intensity of the Alexa Flour 488 image in all nuclei is then measured. The results were then exported to the Columbus 2.4 software (Perkin Elmer) for data analysis. Telomere length distribution and median telomere length were calculated by Life Length’s proprietary algorithms. Statistical analysis of the data was performed using Student’s *t*-test.

## Figures and Tables

**Table 1:** Viability and recovery of the LiPSC (Lonza induced pluripotent stem cells) lines after long-term cryopreservation.

LiPSC Line	Total Viable Cells/mL	% Viability	Resuspension Volume (mL)	Total Viable Cells/Vial	Total Viable Cells Frozen	Percent Recovery
LiPSC-18R-P22	1.85 × 10^5^ ^1^	83.3 ^1^	4.4	8.15 × 10^5^ ^1^	1.0 × 10^6^	81.5%
LiPSC-TR1.1-P19	3.29 × 10^5^ ^1^	75.2 ^1^	5.0	1.64 × 10^6^ ^1^	2.0 × 10^6^	82.0%
LiPSC-ER2.2-P15	2.29 × 10^5^ ^1^	81.2 ^1^	5.0	1.15 × 10^6^ ^1^	2.0 × 10^6^	57.5%

^1^ Average of three vials.

**Table 2:** Concentration, viability, and telomere length of the freshly-thawed and passaged iPSCs. The table shows the cell count and viability of the analyzed cells. Telomere analysis technology (TAT) shows the median telomere length and 20th percentile median telomere length (both in base pairs-bp) for each sample as well as the percentage of short telomeres. The latter is defined as the percentage of the telomeres with a length below 3 Kbp (<3 Kbp). All measurements were performed in quintuplicate.

Sample ID	Cell Conc/mL	Viability (%)	Median Length (Base Pairs)	20th Percentile Length (Base Pairs)	Telomeres <3 kp (%)	Coefficient of Variation (CV)
LiPSc-18R (Freshly thawed)	1.0 × 10^6^	70	13,489	9370	1.44	1.45
LiPSC-18R (Thaw+15 passages)	2.0 × 10^6^	71	13,142	8850	2.14	2.15

## Supplementary Materials

Supplementary materials can be found at https://www.mdpi.com/1422-0067/21/1/108/s1.
