# Supplementary material for: Long-Term Stability and Differentiation Potential of Cryopreserved cGMP-Compliant Human Induced Pluripotent Stem Cells
Source: Int J Mol Sci. 2019 Dec 23;21(1):108. doi: 10.3390/ijms21010108 (PMC6982271; doi:10.3390/ijms21010108)
Supplement: Supplementary file 1 [file ijms-21-00108-s001.pdf]

International Journal of *Molecular Sciences*

Supplementary Tables, Figures and Videos

Supplementary Table S1: Donor information of the three iPSC lines used in the current study.

Sample ID	M / F	HLA Type	Ethnicity
LiPSC-18R	F	A*02/25, B*18/*44, C*05/*12 DRB1*04/*07,DQB1*02/*03	Caucasian
LiPSC-TR1	F	A*02/*31, B*35/*49, C*07/*15 DRB1*01/*12,DQB1*03/*05	Caucasian
LiPSC-ER2.2	F	A*03/*24, B*27/*27, C*01/*05 DRB1*01/*11,DQB1*03/*05	Caucasian

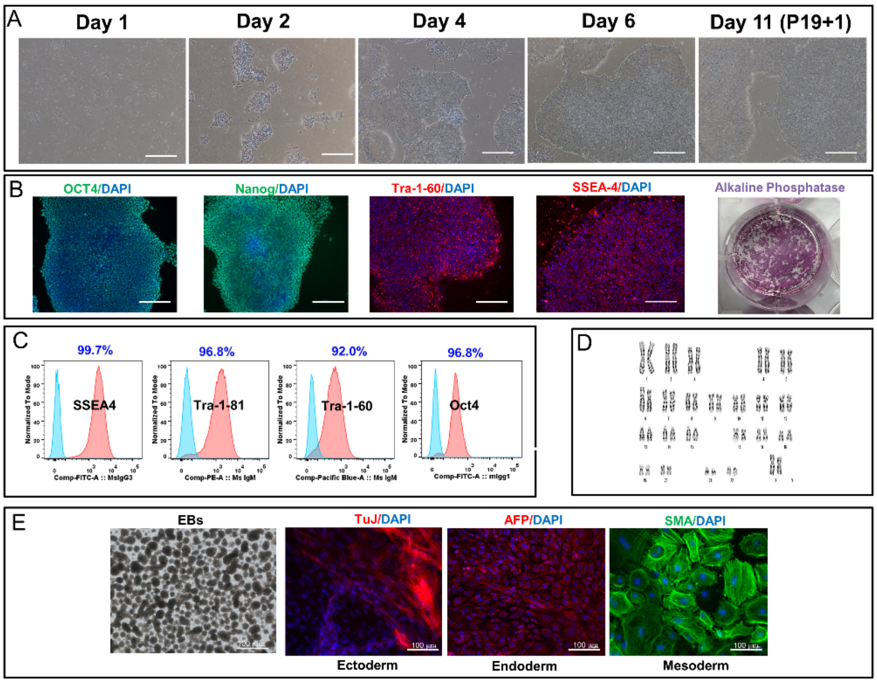

Supplementary Figure S1: Thaw, Expansion, and Characterization of Human LiPSC-Tr1.1 post prolonged cryopreservation. (A) The iPSCs attached and formed typical PSC colonies 1 day post thaw. The cells were passaged on day 6 and formed colonies one passage post thaw. Scale bars, 100µm. **(B)** iPSCs stained positively with OCT4, TRA-1-60, SSEA4, NANOG, and AP. Scale bars, 100µm. **(C)** iPSCs expressing the pluripotent stem cell internal and surface markers OCT4, SSEA4, TRA-1-60, and TRA-1-81 (dark pink). Light blue indicates the isotype control. **(D)** The iPSCs demonstrated a normal karyotype one passage post thaw. **(E)** iPSCs differentiated into embryoid bodies and readily expressing the markers for early ectoderm (TUJ1), endoderm (AFP), and mesoderm (SMA). Scale bars, 100µm.

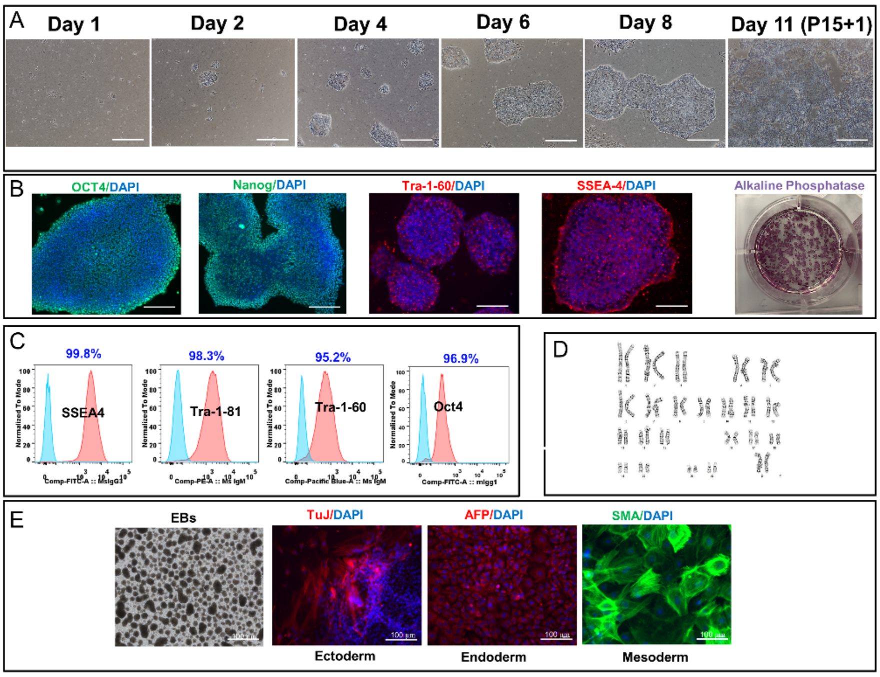

Supplementary Figure S2: Thaw, Expansion, and Characterization of Human LiPSC-ER2.2 post prolonged cryopreservation. (A) The iPSCs attached and formed typical PSC colonies 1 day post thaw. The cells were passaged on day 6 and formed colonies one passage post thaw. Scale bars, 100µm. **(B)** iPSCs stained positively with OCT4, TRA-1-60, SSEA4, NANOG, and AP. Scale bars, 100µm. **(C)** iPSCs expressing the pluripotent stem cell internal and surface markers OCT4, SSEA4, TRA-1-60, and TRA-1-81 (dark pink). Light blue indicates the isotype control. **(D)** The iPSCs demonstrated a normal karyotype one passage post thaw. **(E)** iPSCs differentiated into embryoid bodies and readily expressing the markers for early ectoderm (TUJ1), endoderm (AFP), and mesoderm (SMA). Scale bars, 100µm.

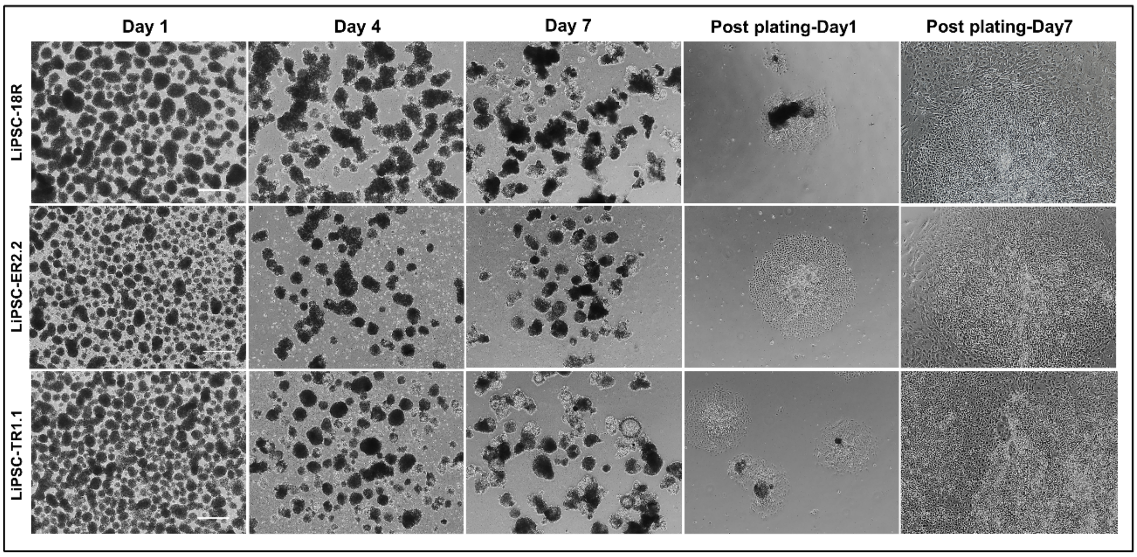

Supplementary Figure S3: Embroid Body (EB) formation of cryopreserved human iPSC lines. LiPSC-18R, LipSC-TR1.1 and LiPSC-ER2.2 formed cell clumps one day post EB induction with various sizes. The clumps attached to gelatin-coated plates on day 7 and grew until day 14. Scale bar: 100µm.

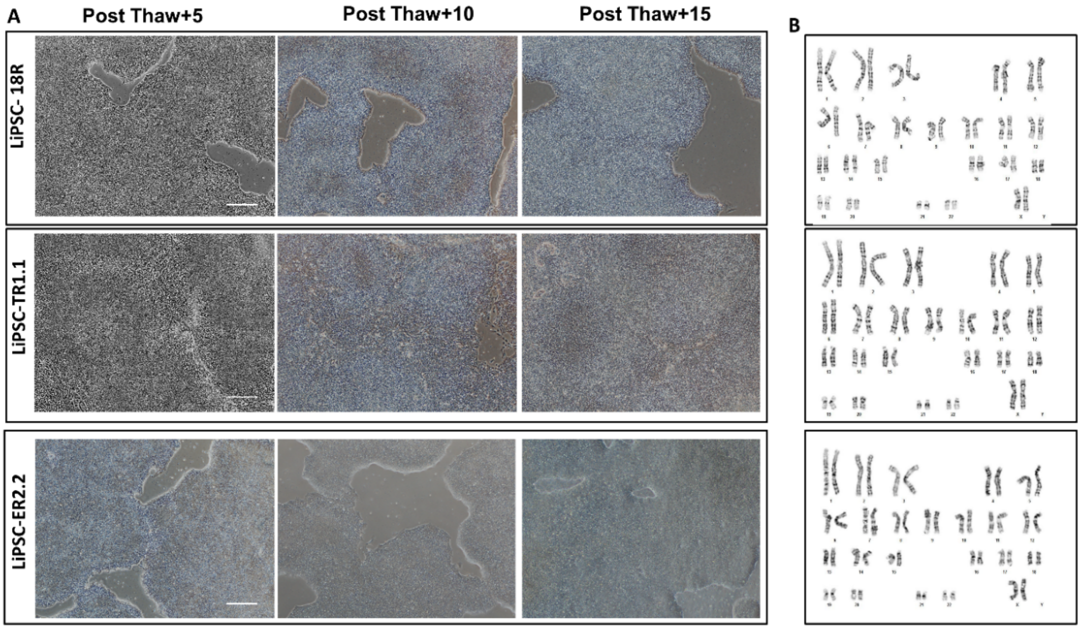

Supplementary Figure S4: Maintenance of LiPSC lines on 2D culture post thaw. (A) All three LiPSC lines were kept undifferentiated for 15 passages on L7™ matrix coated vessels. **(B)** All three lines preserved their genomic stability by showing normal karyotype. Scale bars, 100μm.

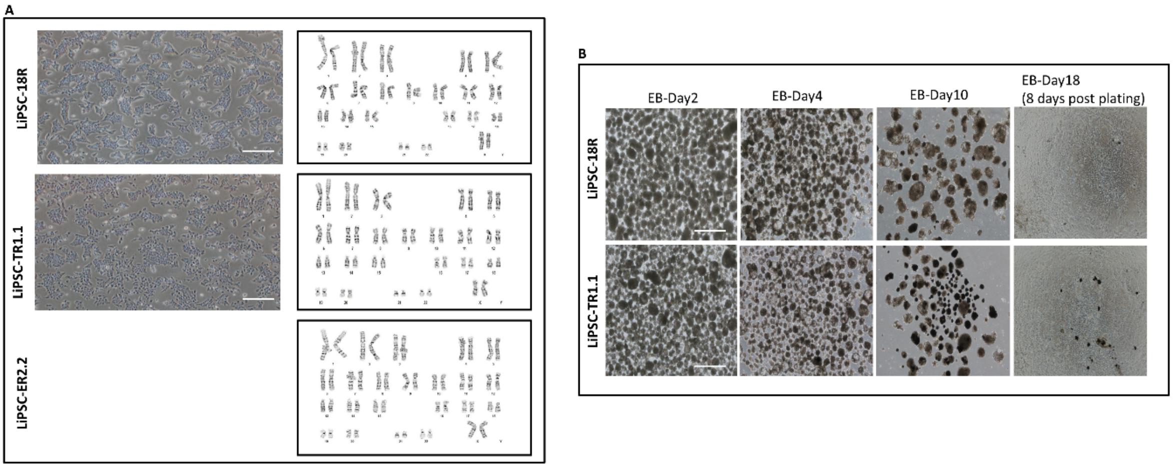

Supplementary Figure S5: Maintenance of LiPSC lines on 2D culture post thaw. (A) All three LiPSC lines preserved their normal phenotype and karyotype post plating and 3D expansion in Biott bioreactor. **(B)** Three LiPSC could form EBs post 3D expansion and expanded as outgrowth after plating on gelatin (Two are shown here). Scale bars, 100μm.

Link to Google Drive Supplementary Video S1 and S2.

https://drive.google.com/open?id=1Bj_hCdwM4ZkEX3TuH8VMyZteuEVg9UX0
